# Enhancing the Conductivity and Dielectric Characteristics of Bismuth Oxyiodide via Activated Carbon Doping

**DOI:** 10.3390/molecules29092082

**Published:** 2024-05-01

**Authors:** Mohamed Khairy, Faisal K. Algethami, Abdullah N. Alotaibi, Rasmiah S. Almufarij, Babiker Y. Abdulkhair

**Affiliations:** 1 Chemistry Department, College of Science, Imam Mohammad Ibn Saud Islamic University (IMSIU), Riyadh 11623, Saudi Arabia; mkomran@imamu.edu.sa (M.K.); falgethami@imamu.edu.sa (F.K.A.); abdullahalotaibi@imamu.edu.sa (A.N.A.); 2 Chemistry Department, Faculty of Science, Benha University, Benha 13518, Egypt; 3 Department of Chemistry, College of Science, Princess Nourah bint Abdulrahman University, P.O. Box 84428, Riyadh 11671, Saudi Arabia; 4 Chemistry Department, Faculty of Science, Sudan University of Science and Technology (SUST), Khartoum P.O. Box 13311, Sudan

**Keywords:** BiOI, activated carbon, activated carbon/BiOI, electrical conductivity, dielectric properties

## Abstract

Activated carbon/BiOI nanocomposites were successfully synthesized through a simplistic method. The produced composites were then characterized using XRD, TEM, SEM-EDX, and XPS. The results showed that BiOI with a tetragonal crystal structure had been formed. The interaction between activated carbon and BiOI was confirmed via all the mentioned tools. The obtained nanocomposites’ electrical conductivity, dielectric properties, and Ac impedance were studied at 59 KHz−1.29 MHz. AC and dc conductivities were studied at temperatures between 303 and 573 K within the frequency range of 59 KHz–1.29 MHz. The 10% activated carbon/BiOI nanocomposite possessed dc and AC conductivity values of 5.56 × 10^−4^ and 2.86 × 10^−4^ Ω^−1^.cm^−1^, respectively, which were higher than BiOI and the other nanocomposites. Every sample exhibited increased electrical conductivity values as the temperature and frequency rose, suggesting that all samples had semiconducting behavior. The loss and dielectric constants (ε′ and ε″) also dropped as the frequency increased, leading to higher dielectric loss. The Nyquist plot unraveled single semicircle arcs and a decreased bulk resistance, indicating decreased grain boundary resistance. Consequently, the electrical characteristics of BiOI, 1C/BiOI, 5C/BiOI, and 10C/BiOI implied their applicability as dielectric absorbers, charge-stored capacitors, and high-frequency microwave devices.

## 1. Introduction

Innovative metal oxide–carbon hybrid materials have garnered significant interest and find use in several fields, such as electronics, energy storage devices, solid-state gas sensors, gas and liquid adsorption, photocatalysis, heterogeneous catalysis, and solar cells [1,2]. Activated carbon (AcC) has extraordinary unique characteristics and excellent surface properties, including pore size, structure, surface area, and chemical and thermal stability [3,4]. These factors made AcC a plausible candidate for gas separation, water treatment, electrode material, supercapacitors, and lithium-ion batteries [5,6]. Moreover, the electrochemical performance of carbon-based electrocatalysts is mostly determined by the characteristics of the carbon support, particularly its electrical conductivity [7]. Several metal oxides (TiO_2_, Fe_2_O_3_, ZnO, WO_3_, and SnO_2_) supported nanoparticles on AcC also may encompass metal hydroxides and oxyhydroxides Ni(OH)_2_, and α-FeO(OH), and metals (Pt, Au, and Ag) [8,9]. Recent articles provided diverse preparations and uses of metal oxides supported on carbonaceous materials [10,11]. The aforementioned carbon-based nanocomposites’ overall chemical and physical properties are significantly influenced by both the interfaces and the grain boundaries in addition to the inherent properties of the individual ingredients [12]. It is commonly known that electrical conductivity has a major role in determining the performance and applicability of these materials as electrode materials in energy storage devices, including fuel cells, lithium-ion batteries, and supercapacitors [13,14,15]. However, several investigations have discovered a relationship between different metal oxides’ electrical conductivity and their capacity for catalysis and gas sensing [16]. Thus, it becomes evident that the exact measurement and comparison of electrical conductivity is necessary for assessing numerous potential applications of carbon-based composites, incorporating nanoparticles of different metal oxides. BiOI, or bismuth oxy-iodide, is a promising active material with a unique layered structure, cheap cost, and semiconducting properties for various electrical and electrochemical applications. The crystal structure is tetragonal, with two slabs of iodide ions ensconced within an open-layered crystal structure of [Bi_2_O_2_]^2+^ layers [17]. Ion diffusion and electron transport are facilitated by the characteristic layered structure. However, BiOI has relatively poor intrinsic electronic conductivity, therefore, creating two-dimensional structures and adding oxygen vacancies are efficient ways to raise electrochemical active sites and enhance electronic conductivity [18]. The wide range of uses for highly electrically conductive nanomaterials has increased their price. These materials are widely used in measuring tools, smart clothing, sensors, cardiopulmonary devices, recorders, wearable monitors, and cardiac monitoring systems. Electroconductive multilayer substrate sensors allow for the continuous monitoring of individuals with cardiovascular conditions. Multiple investigations demonstrated that graphene, carbon nanotubes, and carbon nanoparticles improved electrode layer devices [19].

Conductive materials based on carbon provide new ideas and possible fixes for future technological advancements. These composites are excellent choices because of their desired chemical characteristics, high electrical conductivity, surface area, and thermal conductivity. Carbons and their composites are utilized as current collectors in electrochemical cells (batteries and fuel cells) and electrically conductive components in electrochemical electrodes and conductive inks. These composite materials are extensively used in various fields, such as electronic circuits, wearable electronics, solar cells, and medical diagnostic apparatus [20].

Therefore, in an attempt to improve the electrical properties of BiOI, it will be prepared alongside 1%AC@BiOI (1C/BiOI), 5%AC@BiOI (5C/BiOI), and 10%AC@BiOI (10C/BiOI) nanocomposites. The prepared materials were characterized in several ways; thereafter, the influence of activated carbon dose on the BiOI’s electrical properties will be studied, as well as the effect of the frequency on electrical conductivity, dielectric constant, and dielectric loss.

## 2. Results and Discussion

### 2.1. XRD

**Figure 1:**
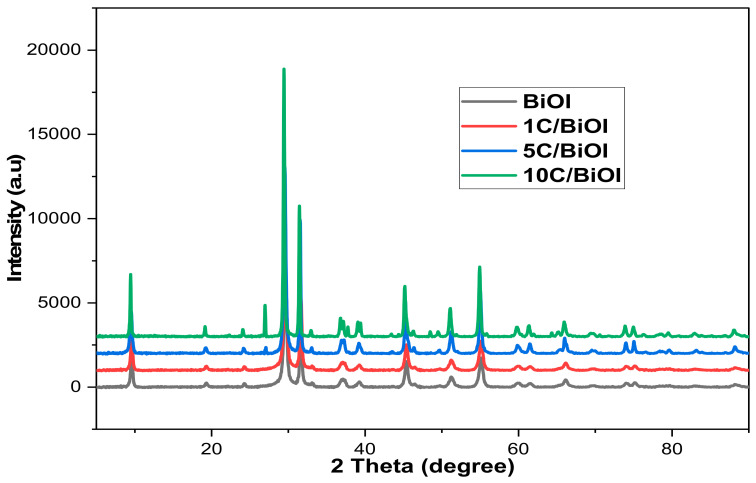
The XRD diffraction patterns of BiOI, 1C/BiOI, 5C/BiOI, and 10C/BiOI.

X-ray diffraction analysis was utilized to look into the phase structure of the BiOl and AcC nanocomposites. Figure 1 displays the diffraction peaks of BiOl and its AcC composites at 2-theta degrees of 9.4, 19.3, 24.1, 31.5, 33.1, 36.6, 39.1, 45.2, 46.4, 49.7, 51.2, 54.9, 61.5, and 66.1, which correspond precisely to the planes (001), (002), (011), (102), (110), (111), (103), (004), (200), (104), (005), and (114) (ICDD 10-0445) [21]. The diffraction pattern provided evidence that the BiOI crystal structure is tetragonal. Additionally, the graphite phase planes (002) and (100) are responsible for the diffraction peaks at 26.7° and 43.5°, respectively, (JCPDS—65-6212) [22,23], and the amplification of those peaks was proportional to the rise in AcC concentration. Additionally, Bragg’s angles were utilized to find out the crystal magnitude via Debye–Scherer’s expression (Equation (1)), while the lattice dimensions (a and c) imperfection were computed via Equations (2) and (3), respectively, and presented in Table 1 [24].

(1) $$D=\frac{0.9\lambda }{\beta \cos\theta }$$

(2) $$a=\frac{\lambda }{\sqrt{3}\;sin\theta }$$

(3) $$c=\frac{\lambda }{sin\theta }$$

D, λ, and β denote the crystal size, wavelength, and the XRD peak width at half-height [24].

Table 1 revealed a change in the main peak towards lower angles when the amount of carbon increases, suggesting a correlation between carbon and BiOI. Furthermore, as the concentration of carbon increases, the crystal’s lattice size (a) slightly decreases while the value of c slightly increases, highlighting the robust interaction between carbon and BiOI.

### 2.2. TEM

**Figure 2:**
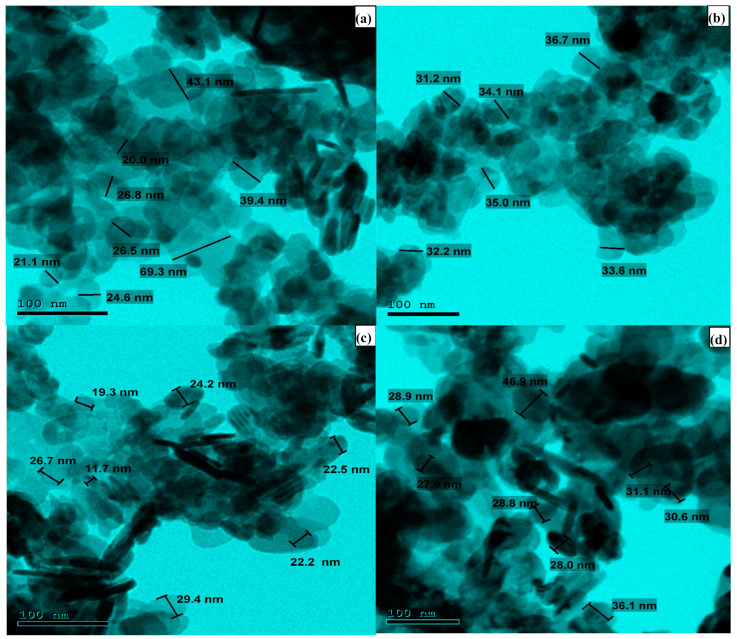
The TEM results for the synthesized (**a**) BiOI, (**b**) 1C/BiOI, (**c**) 5C/BiOI, and (**d**) 10C/BiOI nanocomposites.

**Figure 3:**
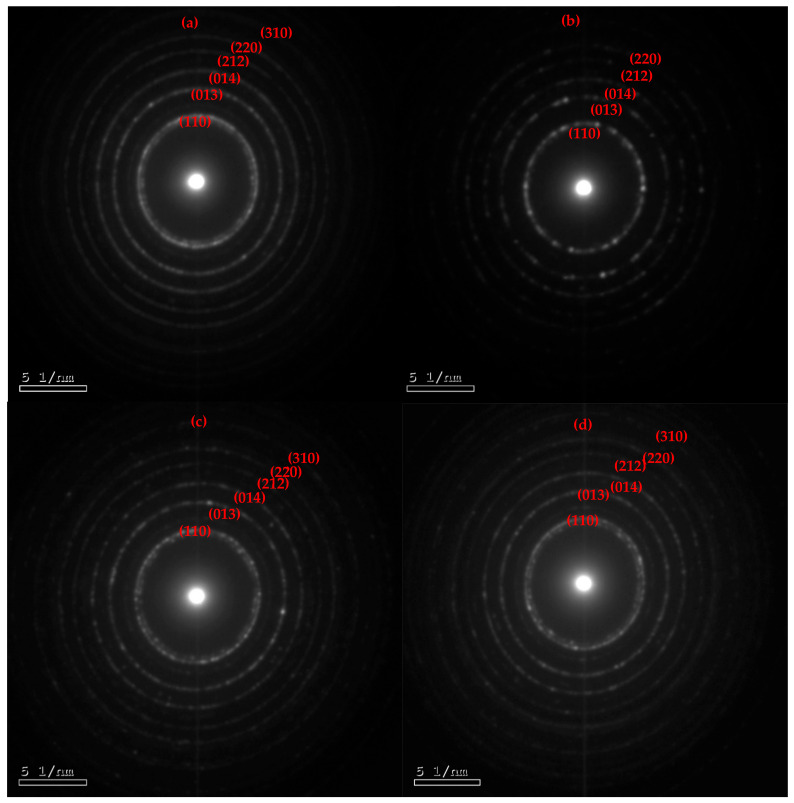
The SAEDs images via electron diffraction HRTEM of the synthesized (**a**) BiOI, (**b**) 1C/BiOI, (**c**) 5C/BiOI, and (**d**) 10C/BiOI nanocomposites.

**Figure 4:**
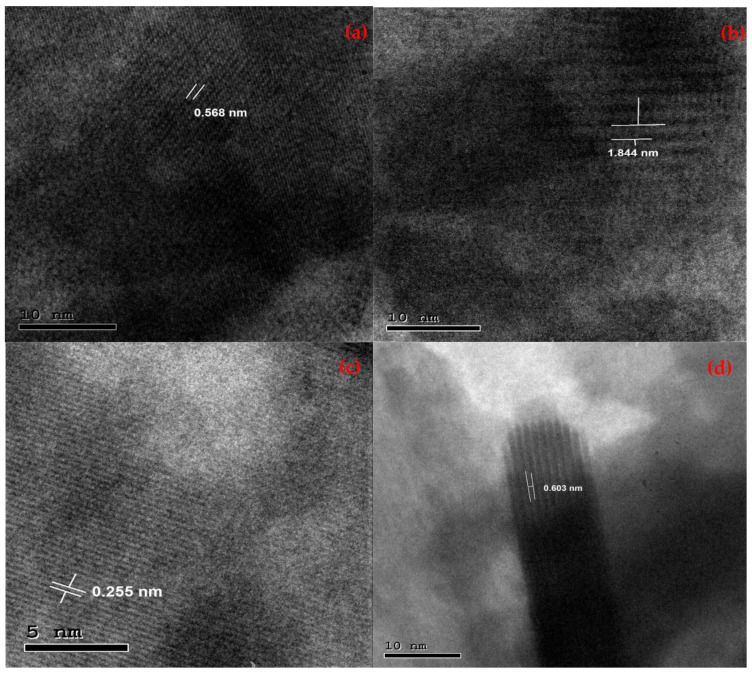
HRTEM d-spacing of the synthesized (**a**) BiOI, (**b**) 1C/BiOI, (**c**) 5C/BiOI, and (**d**) 10C/BiOI nanocomposites.

The HRTEM analysis was employed to inspect the detailed morphology of the BiOI and its AcC nanocomposites and determine their overall particle sizes. Figure 2 revealed particle size ranges of (20.0–69.3 nm), (32.2–36.7 nm), (11.7–29.4 nm), and (26.9–47.9 nm) for the BiOI, 1C/BiOI, 5C/BiOI, and 10C/BiOI, respectively. These results implied that the carbon doping doses served as a disunity agent that downsized the BiOI particles in the composites compared to the undoped BiOI. Those results indicated that the dose of carbon played an intermolecular spacer role since plentiful smaller BiOI nanoparticles were obtained within the nanocomposites. Additionally, selected area electron diffraction (SEAD) was studied for deeper insight into the fabricated BiOI, 1C/BiOI, 5C/BiOI, and 10C/BiOI (Figure 3). The strong juxtaposition between the opaque perimeter and the luminous core of the spheres suggested a particle with hollow BiOI products. The SAED diffraction ring results were aligned with several principal planes revealed via the XRD (Figure 3). Furthermore, the HRTEM image of BiOI, 1C/BiOI, 5C/BiOI, and 10C/BiOI displayed well-defined lattice fringes with interplanar spacings allocated to the planes (110), (001), (302), and (102), respectively (Figure 4). These results were consistent with the tetragonal hollow BiOI nanosphere structures that can be manipulated via varying the Bi, I, and C proportions.

### 2.3. EDX Analysis

**Figure 5:**
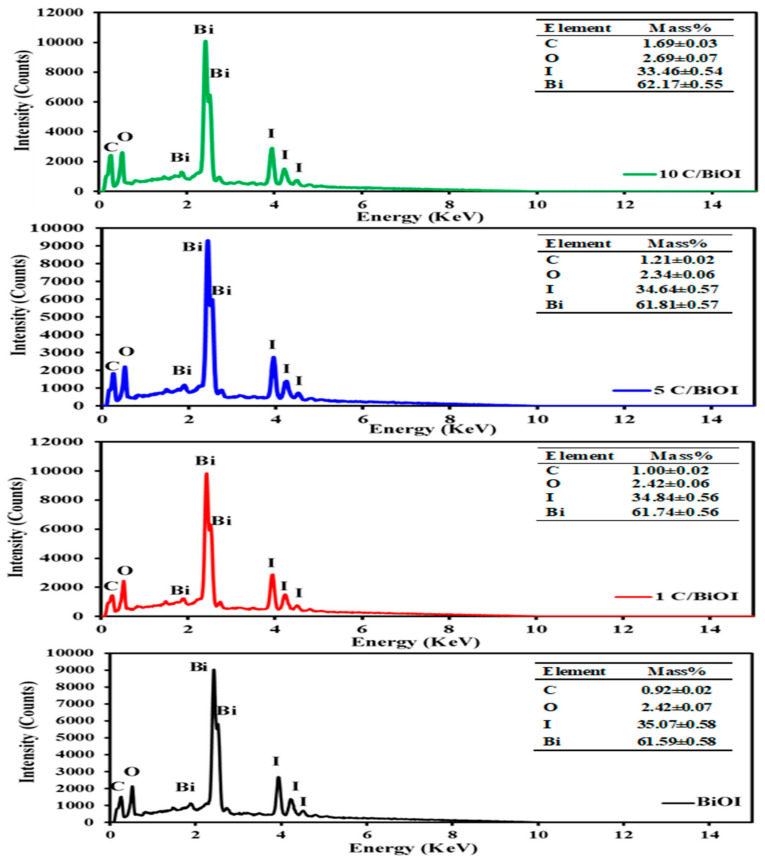
The EDX analysis of BiOI, 1C/BiOI, 5C/BiOI, and 10C/BiOI.

The elemental compositions of the pure BiOI and activated carbon-doped BiOI samples (1C/BiOI, 5C/BiOI, and 10C/BiOI) were evaluated via the EDX results and shown in Figure 5. It was observed that the BiOI sample reveals the presence of Bi, O, and I elements in addition to a minor peak associated with C. All doped samples have several peaks related to Bi, O, I in addition to the appearance of a peak attributed to C with increasing its intensity as the carbon content increases (BiOI; 0.92%, 1C/BiOI; 1%, 5C/BiOI; 1.21% and 10C/BiOI; 1.69%), as shown in the inset tables in Figure 5. The strong interaction between carbon and the BiOI phase could be the cause of this. For complementary and more accurate quantitative results, the compositions of samples are presented in Figure 5.

### 2.4. XPS

**Figure 6:**
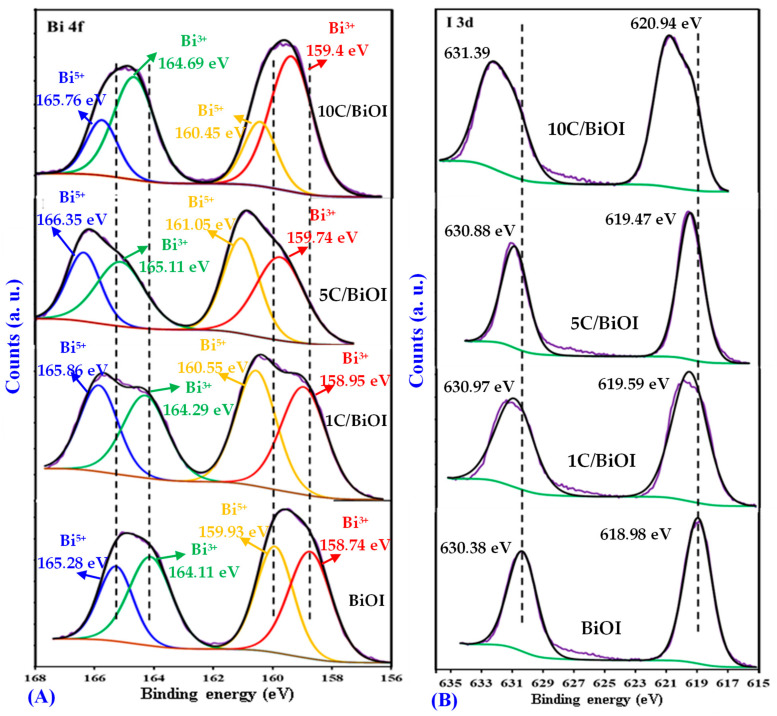
Deconvoluted high-resolution XPS spectra of (**A**) Bi 4f of BiOI, 1C/BiOI/, 5C/BiOI, and 10C/BiOI. (**B**) The I 3d of Bi 4f of BiOI, 1C/BiOI/, 5C/BiOI, and 10C/BiOI.

**Figure 7:**
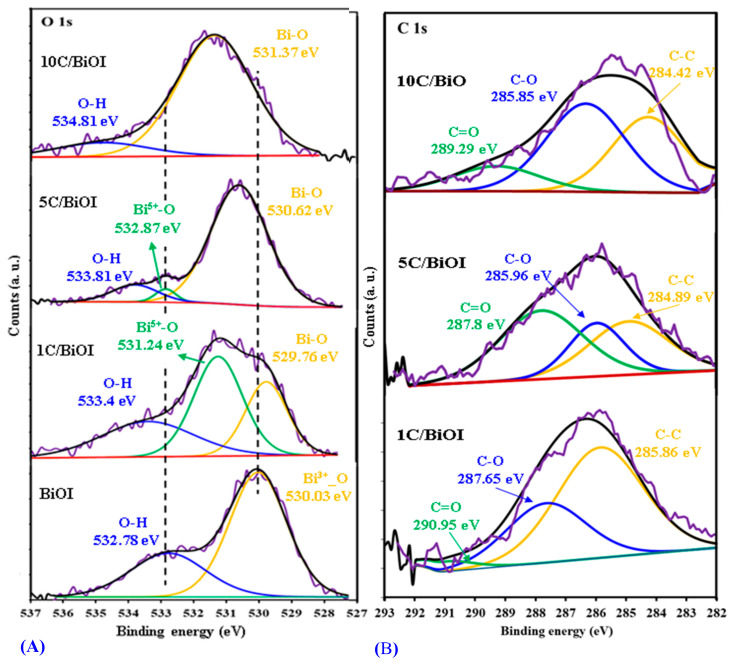
Deconvoluted high-resolution XPS spectra of (**A**) O 1s of BiOI, 1C/BiOI, 5C/BiOI, and 10C/BiOI. (**B**) The C 1s of BiOI, 1C/BiOI/, 5C/BiOI, and 10C/BiOI.

The XPS spectra of BiOI, 1C/BiOI/, 5C/BiOI, and 10C/BiOI were used to evaluate surface elements’ valence state and composition and interaction between the BiOI and carbon added. The XPS magnified spectra of Bi 4f, I 3d, O 1s, and C 1s of all samples were given in Figure 6 and Figure 7. Two diffraction peaks are seen in the Bi 4f spectra of BiOI (Figure 6A) at 159.58 and 164.68 eV, which are attributed to Bi 4f_7/2_ and Bi 4f_5/2_ of [Bi_2_O_2_]^2+^, respectively [25,26,27]. In contrast, the Bi 4f spectrum of 1C/BiOI/, 5C/BiOI, and 10C/BiOI samples exhibit the same two diffraction peaks at (160.38 eV, 165.58 eV), (160.88 eV, 166.18 eV), and (159.78 eV, 164.88 eV), respectively. Importantly, the Bi 4f peak centered at 159.58 eV of all carbon-doped samples slightly shift compared to BiOI (1C/BiOI, 0.8 eV; 5C/BiOI, 1.3 eV; and 10C/BiOI, 0.2 eV), while the peak at 164.68 eV shows a shift (1C/BiOI, 0.9 eV; 5C/BiOI, 1.5 eV; and 10C/BiOI, 0.2 eV), demonstrating how the interaction between carbon and BiOI is modifying the surface chemical environment of Bi [28,29,30]. The peak of Bi 4f_7/2_ (or 4f_5/2_) peak could be decomposed into two bimodal peaks (Figure 6A) for BiOI (158.74 eV and 159.93 eV) and (164.11 eV and 165.28 eV), 1C/BiOI, (158.95 eV and 160.55 eV) and (164.29 eV and 165.86 eV); 5C/BiOI (1159.74 eV and 161.05 eV) and (165.11 eV and 166.35 eV); and 10C/BiOI (159.4 eV and 160.45 eV) and (164.69 eV and 165.76 eV), which could be attributed to Bi^3+^ and Bi^5+^, respectively [31]. These findings suggested the presence of the Bi_3_O_5_I_2_ phase besides the BiOI phase. It was observed that the intensities of the peaks related to Bi^+5^ observed in the spectrum of 10C/BiOI significantly decreased, suggesting a decrease in the Bi_3_O_5_I_2_ phase compared to BiOI. The I 3d spectra of BiOI, as displayed in Figure 6B, have two peaks at 618.98 and 630.38 eV corresponding to I 3d5/2 and I 3d3/2, respectively. These peaks may represent the (−1) oxidation state of the I elements in BiOI [32,33]. The I 3d peaks of 1C/BiOI shift to 619.59, and 630.97 eV, of 5C/BiOI shift to 619.47, and 630.88 eV, and of 10C/BiOI shift to 620.94 and 631.39 eV when compared to those of pure BiOI. This shift may be caused by the interaction of BiOI with carbon.

The O1s spectra (Figure 7A) of BiOI 1C/BiOI/, 5C/BiOI, and 10C/BiOI at 530.03 eV, 529.76 eV, 530.62 eV, and 531.37 eV, respectively, are attributed to Bi^3+^-O bonds of [Bi_2_O_2_]^2^ existing in BiOI [26,34,35]. The O1s spectra (Figure 7A) of BiOI, 1C/BiOI/, 5C/BiOI, and 10C/BiOI at 532.78 eV, 533.4 eV, 533.81, and 534.81 eV, respectively, are attributed to O-H bonds on the surface of BiOI. Furthermore, the peaks at 531.24 eV and 532.87 eV of 1C/BiOI and 5C/BiOI, respectively, correspond to the Bi^5+^-O bonds in the Bi_3_O_5_I_2_ lattice [32,33]. These peaks disappeared in 10C/BiOI, which could be attributed to decreasing Bi_3_O_5_I_2_ phases, as confirmed by the XPS spectrum of Bi 4f of 10C/BiOI (Figure 6A). It was observed that, by increasing the carbon content, there are somehow shifts in all peaks of O 1s (Figure 7A), demonstrating the carbon and BiOI interaction.

The C1s spectra (Figure 7B) observed in 1C/BiOI can be divided into three different peaks. The binding energies at 285.86, 287.65, and 290.95 eV correspond to C-C, C-O, and C=O. The absence of a minor peak in the C 1s spectrum at a lower binding energy of roughly ≈ 281–283 eV [36,37] and the absence of two peaks in the Bi 4f spectrum at ≈ 157 and 162 eV [38,39], respectively, indicating that there is no Bi-C bond. On increasing carbon content, the C 1s peaks of 5C/BiOI shift to 284.89 and 285.96 eV and 287.8 eV and of 10C/BiOI shift to 284.42 and 285.85 eV and 289.29 eV, indicating the surface chemical environment changing of Bi due to the strong interaction between carbon and BiOI [40,41]. From the above results, it can be concluded that there is a good interaction between carbon and BiOI, in addition to the formation of the Bi_3_O_5_I phase beside BiOI [35,42].

### 2.5. DC Conductivity

At temperatures between 303 and 428 K, we used direct current (dc) and alternating current (AC) to examine the electrical characteristics of our samples. When a conducting C/BiOI pellet positioned between two inert electrodes, its entire length experiences a unidirectional electric charge flow, known as dc conductivity. The AC approach differentiates between various mechanisms that contribute to a material’s overall conductivity response, such as grain conduction, grain boundary conduction, and electrode response, in contrast to the DC method, which gives a sample’s global response.

**Figure 8:**
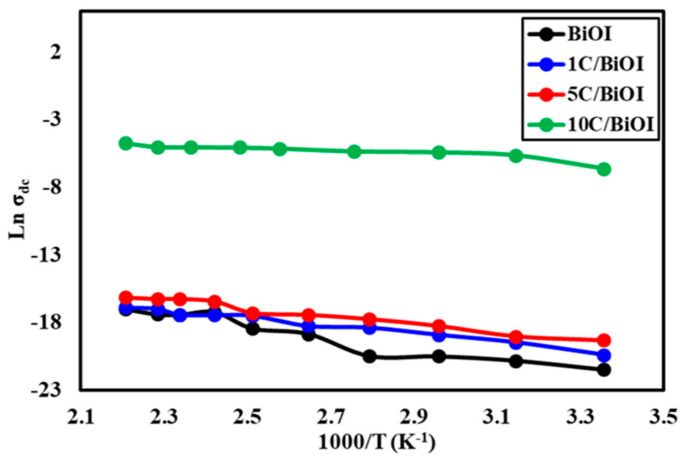
Effect of temperature on the dc-conductivity of BiOI, 1C/BiOI, 5C/BiOI, and 10C/BiOI nanocomposites.

Figure 8 shows the temperature dependency of dc conductivity (σ_dc_) for every sample. The conductivity increases as the temperature increases, indicating the semiconducting behavior. The electrical conductivity followed the Arrhenius equation (Equation (4)), which governs electrical conduction.

(4) $$\sigma_{dc}=\sigma_{o,\;dc\;}\exp\left(\frac{E_{a,dc}}{K_{b}T}\right)$$

Here, σ_o_, dc stands for the pre-exponential factor, E_a,dc_ for the activation energy under the dc field, and K_b_ for Boltzmann’s constant. Table 2 lists the dc conductivity values that were determined by calculating the slope of the linear fit of Arrhenius plots. The conductivity increases in 10C/BiOI > 5C/BiOI > 1C/BiOI > BiOI. Ea ranged from 0.011 to 0.037 eV for all samples. The 10C/BiOI has the lowest activation energy, indicating the ease with which electric current is passed through, thus increasing its electrical conductivity.

### 2.6. AC Conductivity

#### 2.6.1. Effect of Temperature on AC Conductivity

**Figure 9:**
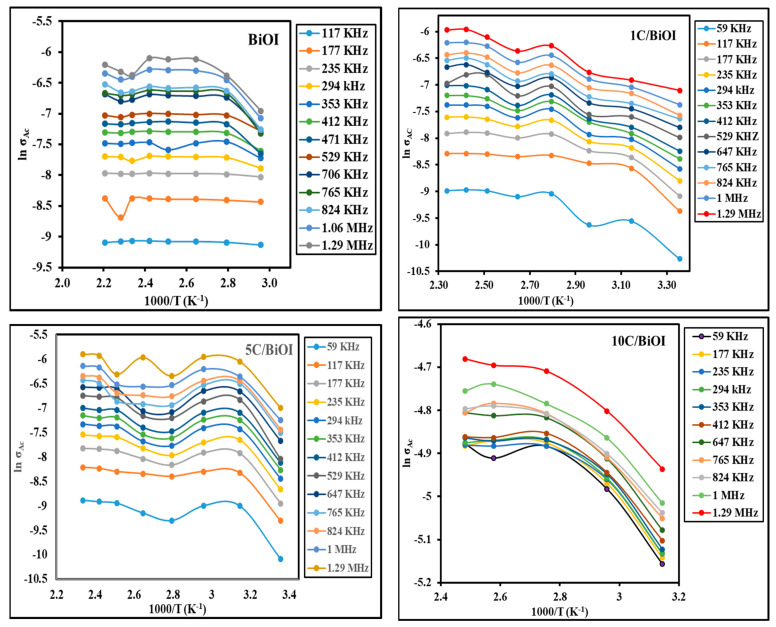
Effect of temperature on the AC conductivity at various frequencies of BiOI, 1C/BiOI, 5C/BiOI, and 10C/BiOI nanocomposites.

Figure 9 shows the plots of lnσ_ac_ vs. 1000/T at chosen frequencies. All the samples demonstrated how conductivity rose with temperature. This does not suggest a rise in charge concentration but is explained by the greater mobility of charge carriers [43,44]. The slopes for straight lines follow the following relation Equation (5):

(5) $$\sigma_{ac}=\sigma_{o,\;AC\;}\exp\left(\frac{E_{a,AC}}{K_{b}T}\right)$$

where σ_o,AC_ is specific conductivity and E_a,AC_ is the ac activation energy.

The conductivity was found to rise as the temperature rose, indicating the semiconductor behavior of all samples. The activation energy was calculated and is presented in Table 2 at selected frequencies.

#### 2.6.2. Effect of Frequency on AC Conductivity

**Figure 10:**
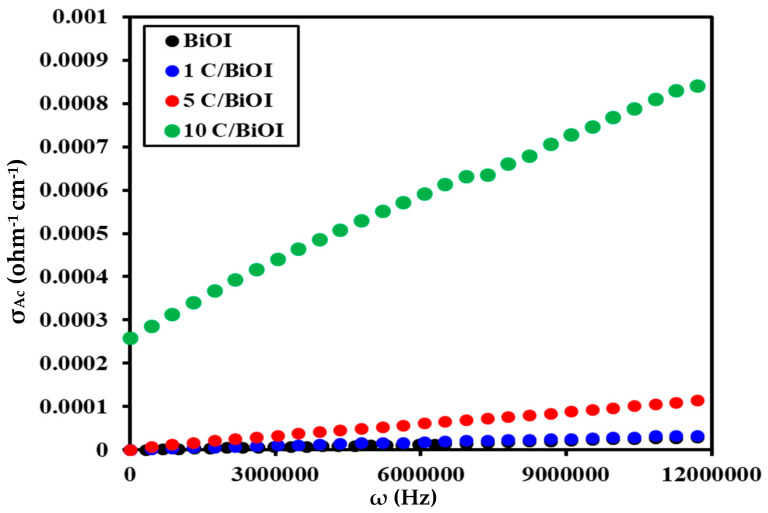
The frequency influences the AC conductivity of BiOI, 1C/BiOI/, 5C/BiOI, and 10C/BiOI nanocomposites.

The AC approach differentiates between various mechanisms contributing to the total conductivity of a material, such as grain conduction, grain boundary conduction, and electrode response. Figure 10 displays the frequency dependence of AC conductivity for the materials under investigation at room temperature. The following relation is used to calculate each sample’s ac conductivity (Equation (6)) [45]:

σ_ac_ = ε′ε_0_ω tan δ (6)

where ε_0_: vacuum permittivity, ε′: dielectric constant, and Tan δ: the loss tangent.

Figure 10 demonstrates that the AC conductivity of all samples rises as the frequency increases, suggesting a semiconducting behavior in line with tiny polaron hopping [46]. Materials with conduction bands originating from unoccupied or “f” orbitals create small polarons. Table 2 summarizes the AC conductivity results. The liberation of charge carriers trapped in confined spaces may cause an increase in conductivity levels at high frequencies, the impact of the applied field’s force, and the heightened migration and movement of the liberated charge carriers among various locations. Freed charge carriers and electron mobility among many metal ions are important factors in the conduction behavior of the material [47].

The AC conductivity measurements in Table 2 and Figure 10 demonstrate how carbon doping contributes to higher conductivity levels. The conductivity value was increased by increasing the carbon amount reach to 255 times for 10C/BiOI samples (2.86 × 10^−4^ ohm^−1^.cm^−1^) compared to pure BiOI samples. In order to preserve system neutrality, carbon doping causes oxygen (IV) atoms to replace oxygen (II) atoms, resulting in oxygen defects [48,49]. As a result, the rise in doping concentration causes an increase in oxygen defects. These native point defects function as shallow donors, enhancing wettability, raising carrier density (enhancing conductivity), and revealing additional active sites.

Creating these neutral defects lowers the height barrier of the grain boundary at the interface, facilitating the flow of charge carriers and increasing conductivity. Moreover, it is thought that carbon doping promotes the development of a more efficient charge-transfer system [50], enhancing the conductivity of samples doped with carbon. The significant variation in the AC conductivity values of 10C/BiOI compared to other samples may also be attributed to decreasing the Bi_3_O_5_I_2_ phase as confirmed by XPS results, which reveals a significant decrease in the peaks related to Bi^5+^ (Figure 6A) compared to the other samples, as well as the disappearance of Bi^5+^-O peak in Figure 7A related to 10 C/BiOI. This also indicates a large gap in the electronic state between 10C/BiOI and other doped samples. Materials used in semiconductors have the following frequency-dependent relationship (Equation (7)):

σ_AC_ (ω) = Aω^s^ (7)

where A and s are constants.

**Figure 11:**
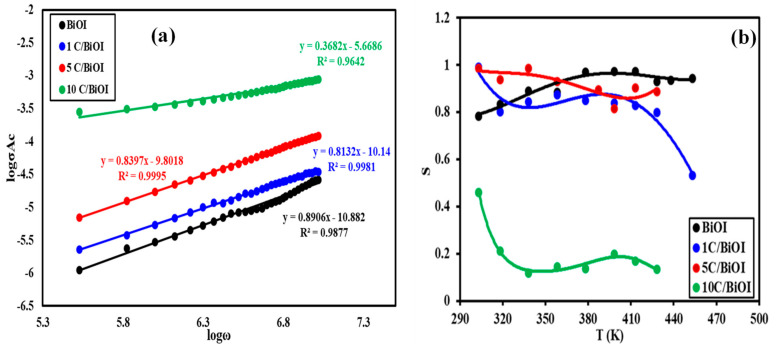
(**a**) AC conductivity frequency dependence in BiOI, 1C/BiOI/, 5C/BiOI, and 10C/BiOI nanocomposites. (**b**) The effect of temperature on the “s” parameter for all samples.

A sudden hopping of the charge carriers results in translational motion if s < 1, while a localized hopping of the species is indicated by s > 1 [51]. The effect is caused by the relaxation resulting from the movement of electrons or atoms by tunneling or hopping between equilibrium locations. The exponent s is determined by graphing the natural logarithm of σ_AC_ (ω) against the natural logarithm of (ω), as depicted in Figure 11a.

The values range from 0.37 to 0.89 (Table 2), indicating that correlated barrier hopping (CBH) is the most likely mechanism in the samples under investigation [52].

In general, the relation between the conduction mechanism and s(T) behavior might suggest a suitable model of the conduction mechanism. According to research, there are two mechanisms for transferring charge carriers (electrons or ions): classical hopping over a potential barrier and quantum mechanical tunneling, or a combination of both [43]. The Correlated Barrier Hopping (CBH) model is the right one when the exponent “s” decreases as the temperature rises [53]. The quantum mechanical tunneling (QMT) model is the most appropriate since the exponent “s” (equal to 0.8) is either slightly increased with temperature or is essentially constant [54].

In order to ascertain the suitable mechanism for the conductivity of the C/BiOI composites, the variation of the exponent “s” concerning temperature is depicted in Figure 11b. It was found that for all carbon-doped BiOI samples, “s” gradually drops with rising temperatures. This behavior agrees with the Correlated Barrier Hopping (CBH) model. On the other hand, the pure BiOI sample shows a steady increase in “s” as the temperature rises, indicating that the best explanation for the conduction mechanism is quantum mechanical tunneling (QMT).

### 2.7. Dielectric Constant (ε′)

**Figure 12:**
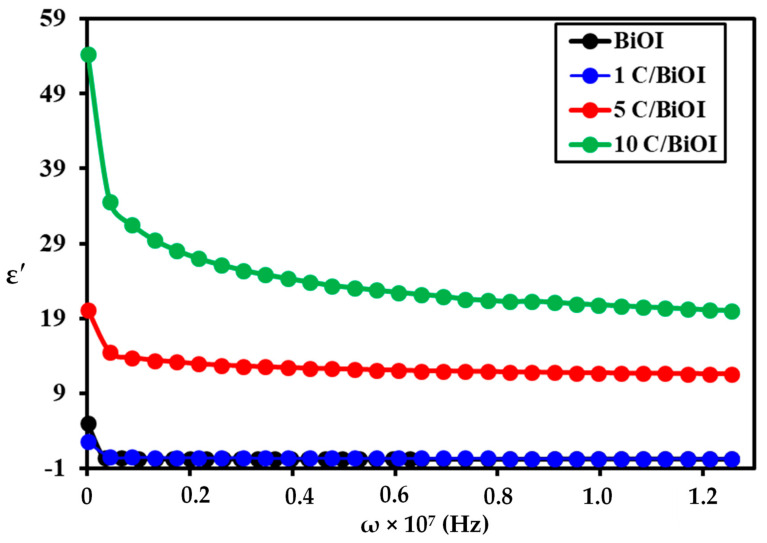
Frequency dependence of dielectric constant in BiOI, 1C/BiOI/, 5C/BiOI, and 10C/BiOI nanocomposites.

The frequency dependence of the ε′ for the examined samples at room temperature is represented in Figure 12, which shows a dispersion in ε′-values as the frequency increases. At lower frequencies, the decline in ε′-values occurs very quickly, whereas at higher frequencies, approaching a constant value takes longer. The dielectric constant exhibits a notable enhancement owing to carbon doping, as observed from the curves. All carbon-doped BiOI samples display higher dielectric constant values (Table 2) at a lower frequency than pure BiOI (7.56 at 70 KHz). As a result of this carbon doping, oxygen defects would result from substituting carbon (IV) atoms for oxygen (II) atoms, throwing off the overall charge of the system and preventing system neutrality. As a result, doping concentration rises, and more oxygen deficiency exists. These native point defects then function as donors [55], who are accountable for enhancing the dielectric conduction process of free charge carriers [48]. Numerous interfaces are created during the doping process, which advances the formation of charge carriers on the interior surface of the C/BiOI lattice and ultimately results in higher dielectric constant values [56]. The high dielectric constant is mostly due to space charge polarization, prevalent in heterogeneous structures of various regions, such as grain and grain borders. The conductivity of the grain is deemed superior to that of the grain border. Because of this, charge carriers experience varying resistances, accumulating charges at the borders and significantly increasing the dielectric constant value [57]. The Maxwell–Weigner model clarifies the dielectric properties of a homogeneous double layer [58]. According to the model, a dielectric material consists of good conducting grains isolated by poorly or resistive conducting borders. Subsequently, as an external electric field is applied, the charge carriers get effortlessly transferred from the grains but will still be assembled at the grain boundaries. Thus, this process creates large polarization and high dielectric constants in semiconductors.

### 2.8. Dielectric Loss Factor (ε″)

**Figure 13:**
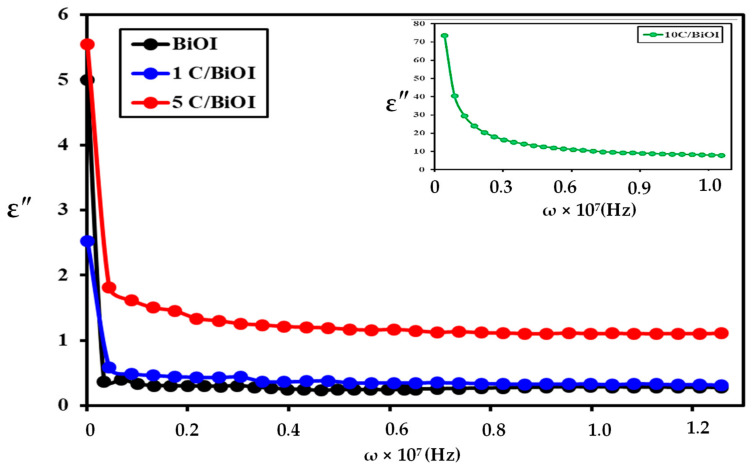
The frequency–dielectric loss (ε″) correlation for BiOI, 1C/BiOI/, 5C/BiOI, and 10C/BiOI nanocomposites.

The dielectric loss specifies the quantity of energy lost due to charge carrier movement. The fluctuation of ε″ as a function of frequency at room temperature is displayed in Figure 13. The behavior obtained is comparable to that of the real part of the dielectric constant, i.e., it decreases as the frequency increases. The ε″-value rapidly drops in the low-frequency region while it remains low in the high-frequency region. This trend can be elucidated by the fact that in the low-frequency region where the samples possess higher resistivity (because of the grain boundary) [59], additional energy is needed to hop the charges between the cations; thus, the loss is high. In the high-frequency region, where the samples have low resistivity (due to grains), less energy is involved in hopping the charges between the cations at the octahedral sites. The polarization of space charges may potentially cause a decrease in dielectric loss as the frequency increases [60].

### 2.9. Impedance Spectroscopy

**Figure 14:**
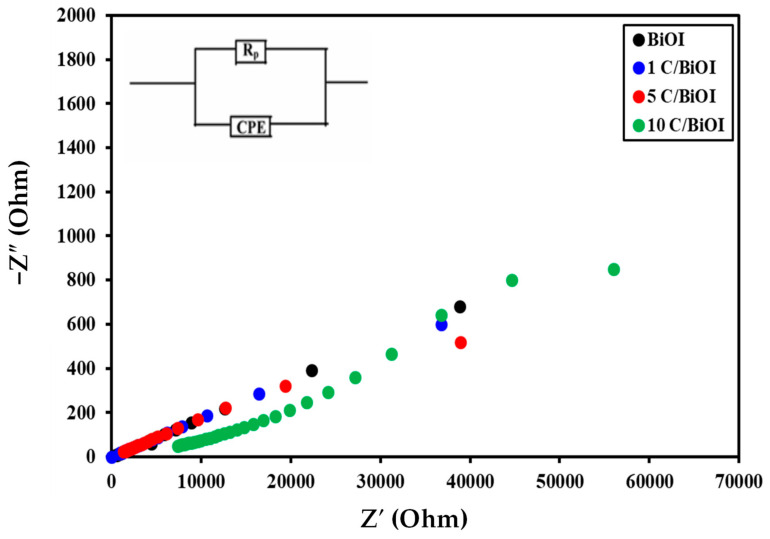
Nyquist (Z″-Z′) plots of BiOI, 1C/BiOI, 5 C/BiOI, and 10C/BiOI at room temperature.

Impedance spectroscopy is a practical and effective method for establishing the relationship between the electrical characteristics and microstructures of substrates [61]. Generally, the electrochemical impedance spectroscopy (EIS) spectra show distinct semicircles in complex impedance planes as they possess diverse relaxation times. The impedance responses caused by the grain border and the grain itself may overlap if the variation in time constants among the processes is less than one hundred. Figure 14 demonstrates the complex impedance spectra for the investigated samples, from which it can be observed that the complex impedance spectra for BiOI and 1C/BiOI samples show only one semicircular arc corresponding to the grain boundary, suggesting that the resistance at the grain boundary is quite high in each sample. The increased surface-to-volume ratio, growing porosity, and greater disorder atomic arrangement could cause the high value of the grain boundary resistance. Instead of showing whole semicircles, the data in the impedance graphs show half semicircles. Since the center of semicircles lies below the abscissa axis, all samples exhibit non-Debye-type dielectric relaxation. The semicircle radius differs because the pure BiOI and 1C/BiOI samples have different resistances [62,63,64]. On the other hand, on increasing the carbon content to 5% and 10%, the semicircle almost disappeared, and only inclined lines were observed, suggesting that only resistive component of the 5C/BiOI and 10C/BiOI composites exists [42]. As observed from Table 1, the resistance of doped samples is lower than that of pure BiOI. The resistance decreases with increasing activated carbon content, which indicates carbon’s effective role in increasing the conductivity of 5C/BiOI and 10C/BiOI nanocomposites. The electrical conductivity of 10C/BiOI nanocomposites with the highest conductivity was compared with the electrical conductivity of other systems and is presented in Table 3. It was found that our composite possesses higher conductivity than others.

## 3. Experimental

### 3.1. Materials

Ethylene glycol (EG) was provided from Sigma Aldrich (Burlington, MA, USA), and bismuth nitrate pentahydrate (Bi(NO_3_)_3_.5H_2_O) was purchased from BDH, London, UK. Potassium iodide (KI) was supplied from Sharlau-Barcelona, Spain, and the activated carbon was provided from Fluka, Buchs, Switzerland (99.9%).

### 3.2. Synthesis of BiOI and Activated Carbon/BiOI Composites

BiOI was synthesized by an in situ method by dissolving equimolar quantities (0.0288 mol) of (Bi(NO_3_)_3_.5H_2_O), and the same moles of KI were dissolved separately in 150 mL and 30 mL of EG, respectively. The EG solutions were heated to approximately 100 °C, after which the two solutions were poured into 200 mL of boiling distilled water (DW), which was subsequently chilled to the ambient temperature, and the product was filtered using a suction system. The procedure was replicated by adding 0.1018, 0.5305, and 1.12 g of AcC to the bismuth solution to obtain 1C/BiOI, 5C/BiOI, and 10C/BiOI.

### 3.3. Characterization

The X-ray diffractometry (XRD) was studied to identify the crystallinity of samples employing Japan-made JDX-8030 X-ray, JEOL (Tokyo, Japan). The patterns used Cu-filtered CuKα radiation (1.5418 Å) driven at 45 kV and 10 mA. The test samples were subjected to ambient temperatures between 2θ = 5 and 80°. TEM-SAED-HRTEM images were used to examine the surface morphologies of the collected samples using a 200 kV speed voltage transmission electron microscope (Tecnai G20, Hillsboro, OR, USA). Using high-resolution JEOL JEM-6700F equipment in conjunction with electron-dispersive X-ray spectroscopy (EDS), energy-dispersive X-ray spectroscopy (EDX) was used to estimate the elemental composition of materials. After monochromatic Al-Kα irradiation, the binding energy values of the synthesized catalysts were modified using C1s (284.6 eV) and evaluated using a KRATOS-AXIS DLD analyzer, Manchester, UK.

### 3.4. Electrical Measurements

We used a two-probe method to measure the electrical conductivity (EC) of tablets 10 mm in diameter and about 1 mm thick. The tablets were made by pressing powder under a 2 × 10^−3^ kg/cm^2^ pressure. Silver paste is placed on both surfaces of the tablet. The tablets are placed in the oven to remove any moisture. Under typical room conditions, we measured the electrical conductivity, dielectric constant, dielectric loss, and impedance using a programmable automatic LCR bridge (model HIOKI IM 3536, Nagano, Japan) at a fixed voltage (1.0 V) and frequencies between 1000 Hz and 2 MHz. The frequency-dependent complex dielectric function can be expressed via Equation (8) [65]:

ε**(ω) = ε′(ω) − jε″ (ω)(8)

With j = √−1, the imaginary part of the permittivity is represented by ε″, while the real part is represented by ε′. Equations (9) and (10) were used to approximate the values for ε′ and ε″ [65]:

ε′ = Cd/ε°A_s_ (9)

ε″ (ω) = ε′(ω) tanδ(10)

where:

ε° (ε° = 8.86 × 10^−12^ F/m): the free space permittivity; d: Thickness; A_s_: cross-section-area; Tan δ: dissipative factor; ω (2πf): electric field frequency. The electrical conductivity at direct (dc) and alternating current (AC) was measured at temperatures ranging from 25 to 180 °C.

## 4. Conclusions

A facile one-pot method was adopted for preparing BiOI, 1C/BiOI, 5C/BiOI, and 10C/BiOI nanomaterials. XRD analysis unraveled the BiOI tetragonal crystal structure in all fabricated products. The electrical characteristics of BiOI, 1C/BiOI, 5C/BiOI, and 10C/BiOI nanocomposites were evaluated. Different frequencies were utilized to measure the dielectric constant and AC electrical conductivity. The proportionality of temperature and frequency and correspondence dc and AC electrical conductivity indicated the semiconducting properties for the four synthesized nanomaterials, a phenomenon attributed to the hopping mechanism for all samples except pure BiOI, which shows the quantum mechanical tunneling mechanism depending on the increase in the value of s with increasing temperature. Compared to pure BiOI, the 1C/BiOI, 5C/BiOI, and 10C/BiOI nanocomposites exhibited better AC conductivity, which rose as the AcC increased. Additionally, the dielectric constant έ and the dielectric loss ε″ as frequency increased, and the 1C/BiOI, 5C/BiOI, and 10C/BiOI responses were higher than the pure BiOI. The impedance analysis showed that grain boundaries play a major role in the conduction process at high frequencies, specifically for BiOI and 1C/BiOI samples. The electrical characteristics of BiOI, 1C/BiOI, 5C/BiOI, and 10C/BiOI results nominated these facilely synthesized nanomaterials for several electrical applications, such as dielectric absorbers, charge-stored capacitors, and high-frequency microwave devices.

## Figures and Tables

**Table 1:** Crystal lattice, crystallite size, and lattice strain obtained from the XRD data.

Sample	2θ (Degree)	D (nm)	a (Å)	c (Å)
BiOI	29.61	15.08	0.02273	0.60286
1C/BiOI	29.61	15.07	0.02272	0.60284
5C/BiOI	29.55	20.93	0.02268	0.60412
10C/BiOI	29.44	34.80	0.02260	0.60633

**Table 2:** The σ_dc_, E_a,dc_, E_a,AC_, s, and A values and the values of σ_ac_, dielectric constant at 70 KHz for the investigated samples.

Sample	s	A	σ_dc_ (ohm^−1^.cm^−1^)	σ_ac_ (ohm^−1^.cm^−1^)	Dielectric Constant		Z (Ohm)	E_a,dc_ (eV)	E_a,AC_ (eV)	
									177 KHz	1.29 MHz
BiOI	0.89	1.31 × 10^−11^	2.23 × 10^−9^	1.1 × 10^−5^		7.56	156 × 10^3^	0.037	0.005	0.057
1C/BiOI	0.81	7.24 × 10^−11^	1.09 × 10^−9^	2.3 × 10^−5^		8.50	130 × 10^3^	0.025	0.050	0.106
5C/BiOI	0.84	1.58 × 10^−10^	3.47 × 10^−8^	7.1 × 10^−5^		14.47	85 × 10^3^	0.026	0.011	0.008
10C/BiOI	0.37	2.15 × 10^−6^	5.56 × 10^−4^	2.86 × 10^−4^		34.55	52 × 10^3^	0.011	0.059	0.050

**Table 3:** Comparison of electrical conductivity of 10C/BiOI nanocomposites with other systems.

Sample	σ_ac_ (ohm^−1^.cm^−1^)	Ref
BiOI thin films	0.125 × 10^−4^	[1]
BiOI thin films at 350 °C	1.524 × 10^−4^	[1]
C900	3.28 × 10^−4^	[2]
Cr, N-codoped TiO_2_	3.05 × 10^7^	[3]
Bismuth oxide/activated (1:1)	1.24 × 10^−5^	[4]
Pure Bi_2_O_3_	1.55 × 10^−7^	[8]
Rice husk activated carb	8.17 × 10^−5^	[4]
8 mmol Bi/CA	4.91 × 10^−3^	[4]
Bi/Commercial CA	0.905 × 10^−7^	[5]
Bi/Rice husk CA	2.59 × 10^−7^	[5]
Commercial activated carb	0.741 × 10^−7^	[5]
10C/BiOI	2.86 × 10^−4^	This work

## Data Availability

Data will be made available upon request.
